# Mit RheMIT können Rheumazentren an der bundesweiten Kerndokumentation teilnehmen – Erweiterung der rheumatologischen Langzeitdokumentation

**DOI:** 10.1007/s00393-023-01373-y

**Published:** 2023-06-06

**Authors:** Johanna Callhoff, Martin Feuchtenberger, Kirsten Karberg, Uta Kiltz, Martin Aringer, Xenofon Baraliakos, Theresia Muth, Anne C. Regierer, Jutta G. Richter, Katja Thiele, Silke Zinke, Katinka Albrecht

**Affiliations:** 1 Programmbereich Epidemiologie und Versorgungsforschung, Deutsches Rheuma-Forschungszentrum Berlin, Charitéplatz 1, 10117 Berlin, Deutschland; 2 Institut für Sozialmedizin, Epidemiologie und Gesundheitsökonomie, Charité – Universitätsmedizin Berlin, Berlin, Deutschland; 3 Med. Versorgungszentren Burghausen – Altötting, MED BAYERN OST GmbH, Burghausen, Deutschland; 4 Rheumatologische Schwerpunktpraxis, Berlin, Deutschland; 5 Ruhr-Universität Bochum, Rheumazentrum Ruhrgebiet, Herne, Deutschland; 6 Rheumatologie, Medizinische Klinik und Poliklinik III, Universitätsklinikum und Medizinische Fakultät Carl Gustav Carus, Technische Universität Dresden, Dresden, Deutschland; 7 BDRh Service-GmbH, Grünwald bei München, Deutschland; 8 Klinik für Rheumatologie, Medizinische Fakultät, Universitätsklinikum Düsseldorf, Heinrich-Heine-Universität Düsseldorf, Düsseldorf, Deutschland; 9 Hiller Forschungszentrum Rheumatologie, Medizinische Fakultät, Universitätsklinikum Düsseldorf, Heinrich-Heine-Universität Düsseldorf, Düsseldorf, Deutschland; 10 Rheumatologische Praxis Berlin, Berlin, Deutschland

**Keywords:** Digitale Rheumatologie, Versorgungsforschung, Rheumatologie, Rheumatische Erkrankungen, Langzeitbeobachtung, Digital rheumatology, Health services research, Rheumatology, Rheumatic diseases, Long-term observation

## Abstract

Die Kerndokumentation der Regionalen kooperativen Rheumazentren wurde im vergangenen Jahr auf die Dokumentationssoftware RheMIT umgestellt. Damit können Einrichtungen, die RheMIT bereits für Versorgungsverträge oder andere Forschungsvorhaben nutzen, zukünftig auch an der Kerndokumentation teilnehmen. Erfahrungsberichte aus einer Klinik, einem Medizinischen Versorgungszentrum und einer Schwerpunktpraxis zeigen, wie die Umstellung auf RheMIT von einem bestehenden Dokumentationssystem bzw. eine neue Teilnahme an der Kerndokumentation mit RheMIT umsetzbar ist. Das Team Kerndokumentation am Deutschen Rheuma-Forschungszentrum Berlin (DRFZ) heißt neue teilnehmende rheumatologische Einrichtungen herzlich willkommen.

## Abstract (en)

The national database (NDB) of the German regional collaborative rheumatology centers was switched to the RheMIT documentation software last year. Rheumatology centers that already use RheMIT for care contracts or other research projects can therefore use the software to also participate in the NDB. Experiences from a hospital, a medical care center and a specialist practice show how the changeover to RheMIT from an existing documentation system or a new participation in the NDB with RheMIT can be implemented. The NDB team at the German Rheumatism Research Center in Berlin (DRFZ) welcomes new participating rheumatology centers.

Seit 1993 werden in der bundesweiten Kerndokumentation der Regionalen Kooperativen Rheumazentren am Deutschen Rheuma-Forschungszentrum Berlin (DRFZ) jährlich aktuelle Daten zur Versorgung von Patient:innen mit entzündlich-rheumatischen Erkrankungen in Deutschland gesammelt [1]. Die Kerndokumentation zeigt seit vielen Jahren Entwicklungen in der internistisch-rheumatologischen Versorgung auf. Beispiele für eine erfolgreiche Verbesserung der Versorgungsqualität sind die kontinuierliche Reduktion der Krankheitsaktivität bei rheumatoider Arthritis (RA) [1], der Anstieg der Erwerbstätigkeit [2] und der Rückgang des Einsatzes von Glukokortikoiden [3] sowie der Osteoporose als Komorbidität [4]. Die Kerndokumentation zeigt auch, an welchen Stellen die Versorgungsqualität weiter optimiert werden kann. Eine auffällig geringe Rate an Biologikatherapie bei seronegativer RA [5] und bei älteren Patient:innen [6], suboptimale Verordnung von Physiotherapie [7] und Funktionstraining [3] sowie ein hoher Bedarf an Schmerzmedikation inklusive Opioiden [8] sind hierfür Beispiele. Ein großer Vorteil der Kerndokumentation ist die Erfassung des gesamten Spektrums der entzündlich-rheumatischen Erkrankungen. Dadurch können auch Entwicklungen in der Versorgung seltener Krankheitsbilder wie der entzündlichen Myopathien [9] oder des primären Sjögren-Syndroms [10] untersucht werden. Neben den Angaben der Rheumatolog:innen zur Therapie und zur Krankheitsaktivität liegt ein Schwerpunkt der Kerndokumentation auf der zusätzlichen Erfassung von durch Patient:innen berichteten Daten. Angaben im Funktionsfragebogen (FFbH), dem Rheumatoid Arthritis Impact of Disease (RAID) und der Abfrage zum Wohlbefinden (WHO-5) sind zentrale Bestandteile vieler Analysen [11, 12]. Jedes Jahr wird aus der Kerndokumentation eine Standardpräsentation veröffentlicht, in der aktuelle Zahlen zur Versorgung entzündlich-rheumatischer Erkrankungen zur Verfügung stehen [3].

Diese Auswertungen sind nur möglich durch die zuverlässige Dokumentation möglichst vieler mitwirkender Patient:innen und Rheumatolog:innen in stationären und ambulanten Einrichtungen. Als die Kerndokumentation 2005 auf eine elektronische Dokumentation umgestellt wurde, haben viele Einrichtungen die Mitarbeit bei der Kerndokumentation beendet, da die Erhebung über eine Dokumentationssoftware statt einer papierbasierten Dokumentation nicht immer frei von Schwierigkeiten in der Umsetzung war. Bisher war es möglich, die Daten mithilfe von 4 verschiedenen Dokumentationssystemen (Documed.rh, RheumaDok, EMIL, ARDIS) einzugeben. Das zog aber einen erheblichen Aufwand in der Datenaufbereitung nach sich sowohl im DRFZ als auch bei den dokumentierenden Einrichtungen.

Mit RheMIT steht jetzt ein einheitliches Dokumentationssystem für die Teilnahme an der Kerndokumentation zur Verfügung. RheMIT ist eine medizinische Dokumentationssoftware, die auf der Grundlage der Software EMIL der Firma itc-ms.de (Marburg) im Auftrag des Berufsverbands Deutscher Rheumatologen e. V. (BDRh) für die Rheumatologie weiterentwickelt wurde, um eine leitliniengerechte Versorgung sowohl in der Regelversorgung als auch in Versorgungsverträgen und Forschungsvorhaben einheitlich und krankheitsspezifisch erfassen zu können [13]. EMIL wird als wissenschaftliches Dokumentationssystem seit über 10 Jahren in den Bereichen Diabetologie, Onkologie, Gastroenterologie und Rheumatologie eingesetzt. In Kooperation mit der Deutschen Gesellschaft für Rheumatologie (DGRh), dem Verband rheumatologischer Akutkliniken e. V. (VRA), dem Berufsverband Deutscher Rheumatologen (BDRh) und dem DRFZ wird RheMIT laufend weiterentwickelt, um die Anforderungen der täglichen rheumatologischen Versorgung sowie der Versorgungsforschung bestmöglich abzubilden. In diesem Rahmen wurde auch die Kerndokumentation integriert, um sie als ein zentrales Forschungsvorhaben der Rheumatologie in RheMIT weiterführen zu können. Einrichtungen, die RheMIT bereits für andere Projekte nutzen, können die dokumentierten Angaben auch für die Kerndokumentation exportieren.

## Voraussetzungen für die Teilnahme

RheMIT muss in der Praxis bzw. Klinik installiert werden. Die technische Umsetzung muss individuell auf die Praxis/Klinikumgebung angepasst werden, da RheMIT mit dem Praxisverwaltungssystem bzw. Klinikinformationssystem und der Laborsoftware verbunden werden kann. Detaillierte Angaben zu den Systemanforderungen und zur Installation stehen im Handbuch in Kap. 13 zur Verfügung (https://update.itc-ms.de/rhemit/handbuch.pdf).

Für die Kerndokumentation ist die vom BDRh zur Verfügung gestellte Basislizenz ausreichend. Diese ist für aktiv an der Kerndokumentation teilnehmende Einrichtungen kostenfrei. Mit dem kostenpflichtigen Zusatzmodul RheMIT Plus stehen weitere Funktionen, wie z. B. Arztbriefschreibung und Einfügen von Scans in die Akte, zur Verfügung. Eine ausführliche Beschreibung und Informationen zu den Kosten stehen auf der Seite itc-ms.de. Da Scores wie der DAS28, die Therapieentscheidungen beeinflussen können, mit einem zertifizierten Medizinprodukt berechnet sein müssen, ist zusätzlich die Installation des Moduls RheCORD DOC der Medizinprodukte-Software RheCORD für alle RheMIT-Nutzer verpflichtend. RheCORD besteht aus insgesamt 3 Komponenten. RheCORD DOC ist die in der Praxis oder Klinik installierte Arztkomponente. Über eine Schnittstelle zu RheMIT werden damit die Scores validiert, was eine rechtssichere, medizinproduktkonforme Verwendung der Scores in RheMIT ermöglicht. Diese Funktionen werden aus RheMIT bedient. Die Übergabe der notwendigen Parameter an RheCORD und die Berechnung werden automatisch im Hintergrund ausgeführt, d. h. die Nutzenden müssen sich nicht in eine zusätzliche Programmoberfläche einarbeiten. Für die Verwendung von RheCORD DOC wird eine jährliche Lizenzgebühr je ärztlichem/r Nutzer:in erhoben. Diese liegt zurzeit bei 120 € pro Jahr.

**Figure 1:**
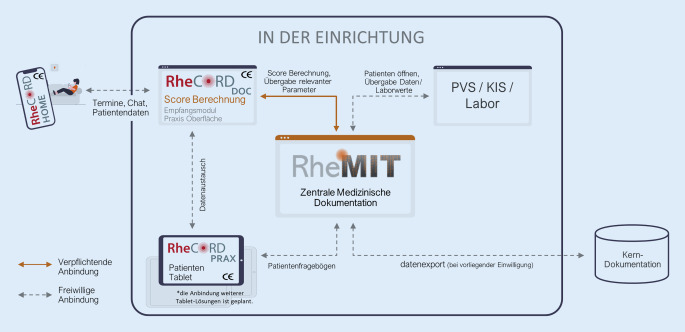

Die Anbindung und Verwendung der Komponenten RheCORD PRAX und RheCORD HOME sind optional und für die Teilnahme an der Kerndokumentation nicht notwendig. RheCORD PRAX ermöglicht es, Fragebögen für Patient:innen mithilfe eines Tablets in der Praxis oder Klinik zu erheben. Dazu wird RheCORD PRAX auf einem Tablet installiert und mit RheCORD DOC verknüpft. Es ist möglich, direkt aus RheMIT heraus auszuwählen, welche Fragebögen für die Patient:innen auf dem Tablet bereitgestellt werden. RheCORD HOME ist eine App für Patient:innen zur eigenständigen Dokumentation ihres Krankheitsverlaufes mithilfe von diagnoserelevanten Fragebögen und Scores sowie ihrer Medikation jenseits der Betreuung in der Klinik oder Praxis. (Abb. 1).

### Erhebung der Items für die Kerndokumentation

Für die Kerndokumentation werden ärztliche Angaben und Fragebögen für Patient:innen erhoben. Erfolgt in RheMIT bereits eine Dokumentation für Selektivverträge oder andere Forschungsvorhaben, ist der ergänzende Aufwand für die Kerndokumentation sehr überschaubar. Neben der obligatorischen Erfassung der Medikation überwiegen optionale Angaben zu nichtmedikamentösen Therapien, Krankenhausaufenthalten, Arbeitsunfähigkeit etc. Diese Items können z. B. durch eine RFA (rheumatologische Fachassistenz) oder eine studentische Hilfskraft dokumentiert oder automatisch aus RheCORD PRAX übertragen werden. Eine Auflistung der obligatorischen und fakultativen Kerndokumentation-Items ist in Tab. 1 dargestellt. Wenn RheMIT bereits installiert ist, kann angezeigt werden, welche Items zur Kerndokumentation gehören.

**Table 1:** 

Ärztliche Angaben	Fragebogen Patient:in
*Obligatorisch* Erhebungsdatum Geburtsjahr, Geschlecht Rheumatologische Hauptdiagnose (ICD 10) Basistherapie (Dosis, Intervall, Seit‑, Bis-Datum, Absetzgrund) Andere medikamentöse Therapien (Glukokortikoide, NSAR, Analgetika) Krankheitsaktivität Komorbiditäten Bei RA: DAS28 *Optional* Größe, Gewicht Versorgungsmodell (z. B. ASV, Regelversorgung) Zeitpunkt der Diagnosestellung Diagnosesicherheit Krankenversicherung Betreuungsbeginn in der Einrichtung (bei Neupatienten: Früherkennungssprechstunde, Verdachtsdiagnose) Ergänzende Therapien (Krankengymnastik, Ergotherapie, Patientenschulung, Rheumafunktionstraining) Endoprothetik COVID-Erkrankung Bei RA: Rheumafaktor, ACPA; bei axSpA: HLAB27 Bei SLE: ECLAM; bei PsA: DAPSA	*Der Fragebogen soll ausgefüllt werden, alle Angaben sind optional* Beginn rheumatischer Beschwerden Erste rheumatologische Untersuchung Erster Praxisbesuch Schulabschluss, Berufsausbildung Mitglied einer Patientenorganisation Größe des Wohnorts, Entfernung der rheumatologischen Einrichtung vom Wohnort Krankheitsaktivität Gesundheitszustand RAID WHO‑5 FFbH Bei axSpA: BASDAI, BASFI Morgensteifigkeit Arbeitsunfähigkeit Krankenhausaufenthalte, Rehabilitation Therapiezufriedenheit Erwerbstätigkeit, Berentungsstatus Rauchen

*ACPA* anti-citrullinierte Proteinantikörper, *axSpA* axiale Spondyloarthritis, *DAPSA* Disease Activity in Psoriatic Arthritis Score, *ECLAM* European Consensus Lupus Activity Measurement, *FFbH* Funktionsfragebogen Hannover, *PsA* Psoriasisarthritis, *RA* rheumatoide Arthritis, *RAID* Rheumatoid Arthritis Impact of Disease, *SLE* systemischer Lupus erythematodes, *WHO‑5* Fragebogen zum Wohlbefinden

Dafür müssen in der Übersicht, an welchen Studien/Versorgungsverträgen eine Person teilnimmt, die Kerndokumentation und das Einverständnis zur Teilnahme angekreuzt werden. Bei den Visitengründen werden dann die Items der Kerndokumentation farblich markiert.

## Erfahrungsberichte

### Umstellung von RheumaDok auf RheMIT in einer Praxis (Kirsten Karberg)

Unsere Praxis nimmt seit 2003 an der Kerndokumentation teil. Im Jahr 2020 wurde die Dokumentation auf RheMIT umgestellt. Das Überspielen aller alten Datensätze aus RheumaDok (große Anzahl, da der Dokumentationszeitraum 17 Jahre umfasste) hat ohne Datenverluste sehr gut funktioniert. Allerdings hat das erste Übertragen eine längere Zeit benötigt. Für das Einrichten von RheMIT mit Anbindung von RheCORD DOC und Einrichtung der Tablets wurde eine studentische Hilfskraft eingestellt. Unter dieser Bedingung ist der Umstieg von RheumaDok auf RheMIT in unserer Praxis von einem auf den anderen Tag gelungen.

Mitarbeiter:innenschulung zur Nutzung von RheMIT wurden durchgeführt, und die Anwendung wurde in der Praxis geübt. Wichtig ist dabei, die Praxisabläufe an der Anmeldung immer wieder zu besprechen und auf den Einsatz der digitalen Unterstützungssysteme abzustimmen. Hinzu kommt die Aufklärung der Patient:innen, die sich an die Benutzung eines neuen Tablets gewöhnen müssen.

Die Erhebung der Kerndokumentation gehörte schon immer zu einem Arbeitsschritt in unserer Praxis, insofern war die Hürde für die Mitarbeiter:innen und Patient:innen nicht so hoch, hierfür auch eine komplett digitale Lösung zu akzeptieren. Für mich als dokumentierende Ärztin und für meine Mitarbeiter:innen ist durch die Einführung von RheMIT die Dokumentation im Vergleich zu RheumaDok deutlich einfacher geworden. Die Eingaben sind fast intuitiv und auf einen Blick zu sehen, es benötigt nur noch wenige Mausklicks, um alle erforderlichen Eingaben in einer Visite zu erheben.

Die Labordaten werden automatisch über eine definierte Schnittstelle der Visite zugeordnet. Die Errechnung der Scores erfolgt automatisch, sobald die Laborwerte vorhanden sind, und wird medizinproduktkonform validiert. Über ein zusätzliches Tool der Plausibilitätsprüfung lassen sich schon vor dem Datenexport viele Fehler beheben. Die Nachfragen durch das Kerndokumentationsteam im DRFZ sind deutlich weniger geworden. Somit können wir die Anforderungen der Dokumentation für die Regelversorgung, für Selektivverträge und nun auch für die Kerndokumentation in einem System erfüllen.

Im Laufe der vielen Jahre haben wir verschiedene Varianten der Dokumentation von Papier über Papier- und digitale Fragbogenerfassung bis jetzt zur komplett digitalen Erfassung kennengelernt. RheMIT vereinfacht diese Anforderungen auf eine sehr pragmatische Weise, ist sehr übersichtlich, einfach in der Bedienung, flexibel auf individuelle Praxisbedürfnisse anzupassen und vermeidet die Doppeldokumentation. RheMIT ist für unsere Praxis eine sehr innovative Lösung, die uns hilft, Zeit bei der notwendigen Dokumentation einzusparen und dafür mehr für den direkten Kontakt mit den Patient:innen zu gewinnen.

### Nutzung von RheMIT an einer Universitätsklinik (Uta Kiltz)

Als Universitätsklinik mit dem Forschungsschwerpunkt auf der axialen Spondyloarthritis stand die standardisierte Erfassung von klinischen Daten und Assessments schon lange im Fokus unseres Interesses. Daher planten wir ab 2018 den Einsatz von einem breit verfügbaren Tool, welches neben den klinischen Daten auch die Möglichkeit bot, diverse Krankheitsausprägungen mit standardisierten Messinstrumenten zu erfassen. Für das lokale Register LORE (*L*angzeitbe*o*bachtung von Patient:innen mit der Diagnose einer entzündlich-*r*heumatischen *E*rkrankung) wurde 2019 mit dem aktiven Einschluss der Patient:innen begonnen und die Datenbank 2020 in RheMIT überführt. Parallel zu der Überführung in RheMIT haben wir uns entschieden, auch an der Kerndokumentation des DRFZ teilzunehmen, und haben 2021 mit der Datenerhebung begonnen.

Alle ambulanten Patient:innen mit der Diagnose einer entzündlich rheumatischen Erkrankung werden bei der ersten Vorstellung im Rheumazentrum Ruhrgebiet gefragt, ob sie der standardisierten Datenerfassung zustimmen. Mit Zustimmung unterschreiben sie die Einwilligungserklärung für das LORE-Register und die Kerndokumentation. Die Patient:innen erhalten zu der Baseline-Visite einen Fragebogen zu demografischen und klinischen Variablen und ein krankheitsspezifisches Assessment. Der Fragebogen umfasst sowohl Variablen des LORE-Registers als auch die Daten der Kerndokumentation. Die Einwilligung und die halbjährliche Herausgabe der krankheitsspezifischen Assessmentbögen wird im Krankenhausinformationssystem dokumentiert, sodass ein einmaliges Ausfüllen der Registerunterlagen pro Halbjahr pro Patient:in gewährleistet ist.

Dokumentationsassistent:innen übertragen die Daten, die aktuell noch auf Papier dokumentiert werden, in RheMIT. Angestrebt wird eine tagesaktuelle Dokumentation, sodass Rückfragen bei inkonsistenten oder fehlenden Daten unmittelbar erfolgen können. Die Einführung von Tablets ist in Planung, müssen jedoch noch in die IT-Dachstruktur der Klinik implementiert werden.

Die Registerdaten stehen allen hausinternen wissenschaftlich tätigen Kolleg:innen zur Verfügung. Der Datendownload wird durch geschulte Dokumentationsassistent:innen durchgeführt. Durch die Nutzung des kostenpflichtigen RheMIT Plus-Moduls verfügen wir über die Möglichkeit, gezielte Abfragen durchzuführen (z. B. zur Prävalenz der RA in unserer Kohorte). Diese Selektionen dienen dann als Basis für Auswertungen und Datenexporte, welche auch als Diagramme dargestellt werden können.

RheMIT bietet eine klare Datenoberfläche sowie leicht zu integrierende Updates, die eine kontinuierliche Verbesserung der Datenerfassung ermöglichen. Interessant sind für uns auch die Anbindung von RheCORD DOC und die damit einhergehende Möglichkeit, durch zusätzliche Anbindung der optionalen RheCORD-Komponenten patient:innenberichtete Daten außerhalb von Präsenzstudienvisiten zu erfassen. Durch die intensive Nutzung von RheMIT entstanden vielfältige Fragen zur Datenstruktur, die in Kooperation mit dem BDRh und dem DRFZ größtenteils gelöst werden konnten.

Im Rheumazentrum Ruhrgebiet gab es im Wesentlichen 2 Herausforderungen: Anbindung von RheMIT an das Krankenhausinformationssystem und die Konfiguration der Auswertedatei. Die Auswertedatei war ohne detaillierte Kenntnis zu Datenexporten und Darstellung der Items nicht zu nutzen, da eine Interpretation der Daten nicht möglich war. Die Anbindung von RheMIT an das Krankenhausinformationssystem besteht aktuell nicht, da parallel zur Nutzung von RheMIT die Umstellung auf ein komplett neues Krankenhausinformationssystem durchgeführt wurde. Aktuell sind wir in Gesprächen mit der IT-Abteilung, um die Anbindung an das Labor und die Nutzung von Tablets zur elektronischen Dokumentation der Assessmentbögen zu gewährleisten. Damit würde ein großer Teil der aktuell noch notwendigen Doppeldokumentation entfallen. Mit der Entscheidung der RheMIT-Nutzung war initial nicht klar, dass zusätzliche Kosten durch die Nutzung des RheMIT Plus-Moduls entstehen.

Die Nutzung eines Dokumentationssystems erfordert, dass genau festgelegt wird, welche Variablen regelmäßig bei den Patient:innen erhoben werden. Durch die prospektive Dokumentation der Daten kann eine unmittelbare Rückmeldung an die Weiterbildungsassistent:innen der Ambulanz über die Qualität der erhobenen Daten erfolgen und somit auch eine Besserung der Datenqualität im Routinebetrieb erreicht werden. Die Bereitstellung der Primärdaten erfolgt inzwischen in verbesserter Qualität. Es sind jedoch noch Fragen insbesondere für die longitudinale Auswertung der Primärdaten zu klären.

### RheMIT an einem MVZ (Martin Feuchtenberger)

RheMIT wurde gezielt für die fachspezifischen Anforderungen in Sachen Dokumentation, Steuerung von Praxisabläufen und Routinevorgängen in der ambulanten Rheumatologie entwickelt. Da in einem medizinischen Versorgungszentrum (MVZ) in der Regel mehrere unterschiedliche Fachgebiete angesiedelt sind, stellt sich zunächst die Frage, ob in einem solch interdisziplinären Konstrukt RheMIT überhaupt zum Einsatz kommen kann. Dies kann klar bejaht werden, da das Bindeglied zwischen den Fachdisziplinen innerhalb eines MVZ in der Regel die Praxisverwaltungssoftware (PVS) ist, über die beispielsweise die Abrechnung u. a. gegenüber der KV erfolgt. RheMIT verfügt über entsprechende Schnittstellen zu den gängigen PVS-Systemen und kann so auch in einem interdisziplinären MVZ betrieben werden.

Die Grundidee von RheMIT war von Anfang an, auf Basis einer benutzerfreundlichen Eingabeoberfläche eine vollständige, effiziente und leitliniengerechte Dokumentation zu ermöglichen. Die Daten werden einmalig in RheMIT erfasst und vielfach verwendet. Dies schließt eine nahezu komplett automatisierte Brieferstellung samt Versand (KIM: Kommunikation im Medizinwesen oder Fax) (Anmerkung: Das ist eine Funktion der kostenpflichtigen RheMIT Plus-Version) sowie den Export von Daten in die Kerndokumentation, in Selektivverträge und in das PVS (Abrechnung) ein. RheMIT verfügt in der Plusfunktion über die Möglichkeit, den eigenen Patient:innenstamm für strategische Überlegungen, für eigene wissenschaftliche Projekte oder auch für eine Machbarkeitsprüfung bei klinischen Studien (Feasibility) auszuwerten. Wichtig ist im Hinblick auf die Datenverwendung, dass die Installation von RheMIT grundsätzlich auf dem lokalen Server der Einrichtung erfolgt und die Daten auch weiterhin ausschließlich im Besitz der Einrichtung verbleiben. Ein Datenexport erfolgt nur projektbezogen und nach Veranlassung der Anwender:in vor Ort. Durch eine deutschlandweit einheitliche Erfassung rheumatologischer Daten in der Routineversorgung können die Daten ohne Mehraufwand im Sinne einer Doppeldokumentation wissenschaftlichen Registern und anderen Projekten zur Verfügung gestellt werden. Dies dürfte zu einer deutlichen Steigerung der Datenqualität, aber auch zu einer erheblichen Ausweitung der Zahl beteiligter Einrichtungen führen. RheMIT bietet eine klare Struktur in der Datenerfassung: Die Felder werden der Reihe nach von den nichtärztlichen und ärztlichen Mitarbeiter:innen bearbeitet. Projektbezogene Prüfungen auf Vollständigkeit und Plausibilität unterstützen bei der Dokumentation. Durch diese einfache, aber verbindliche Struktur haben sich die medizinische Qualität und auch die Mitarbeiter:innenzufriedenheit erhöht. Die komplett digitale Dokumentation in RheMIT hat zudem die konsequente Umsetzung von Home-Office-Arbeitsplätzen in unserer Einrichtung ermöglicht: Die Nachbereitung der Akten, Befundeingangssichtung, Brieffreigabe etc. erfolgen nahezu ausschließlich im Homeoffice. Wiederholt wurde in Gesprächen mit Stellenbewerber:innen klar, welche Bedeutung diese Option für zukünftige Mitarbeiter:innen besitzt und wie sehr dadurch die Attraktivität als Arbeitgeber:in gesteigert werden kann.

Die Installation auf dem lokalen Server verlief unproblematisch. Bei der Erstinstallation müssen die Schnittstellen z. B. zum PVS, der Laborsoftware, zu Sono-GDT (Gerätedatentransfer), Scanner, Drucker etc. einmalig eingerichtet werden. Die einzelnen Arbeitsplätze (Clients) werden innerhalb des Netzwerks lokal angebunden. Der Zeitbedarf hierfür liegt im Bereich eines Tages. Einen kritischen Punkt stellt die Migration von Daten aus anderen Systemen in die Datenbank von RheMIT dar. Für RheumaDok, EMIL und Documed.rh ist dies umgesetzt, bezüglich anderer Systeme muss individuell geprüft werden. Herr Schumann von www.itc-ms.de und sein Team stehen hier für individuelle Fragen zur Verfügung und begleiten den Prozess der Installation (in der Regel mittels Fernwartung). Sofern ein Neustart in einer Praxis, einem MVZ oder einer Ambulanz geplant wird, sollte idealerweise von Anfang an mit RheMIT gearbeitet werden, um das Thema der Datenmigration zu vermeiden. Entscheidend ist eine gute Vorbereitung der Installation durch frühzeitige Einbindung der lokalen IT (Firewall, Sicherungsroutinen etc.) und der Mitarbeiter:innen. Es muss allen im Team bewusst sein, dass eine neue IT-Plattform im Zentrum aller Abläufe steht und damit auch weitere Anpassungen in zahlreichen Prozessen erforderlich sind. Viele SOPs („standard operating procedures“) müssen neu geschrieben werden. Zum Glück handelt es sich hier in der Regel um Vereinfachungen der Abläufe!

## Stand der Kerndokumentation in 2023

**Figure 2:**
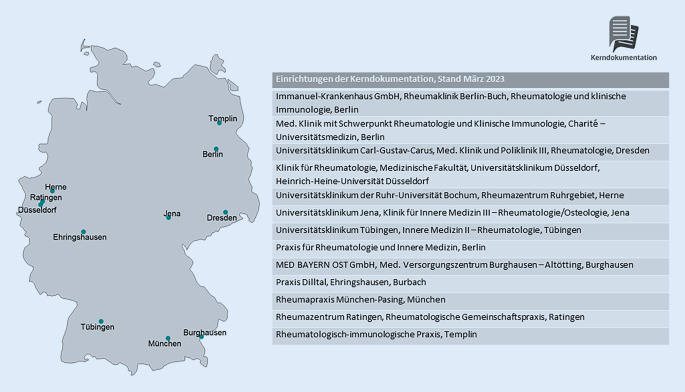

Im März 2023 nehmen 13 Einrichtungen an der Kerndokumentation teil, davon 7 Kliniken und 6 rheumatologische Praxen/MVZ (Abb. 2). Hiervon nutzen 11 Einrichtungen RheMIT.

## Perspektiven für die Kerndokumentation

Durch die Integration der Kerndokumentation in RheMIT können weitere rheumatologische Praxen, MVZ und Kliniken für die Teilnahme gewonnen werden. Für die Versorgungsforschung in der Rheumatologie ist es von großer Bedeutung, die Kerndokumentation langfristig zu erhalten und eine möglichst repräsentative Erhebung für ganz Deutschland zu gewährleisten. Teilnehmende Einrichtungen können Ideen für wissenschaftliche Auswertungen in das jährliche Kerndokumentationstreffen einbringen. Derzeit bemühen wir uns, die Voraussetzungen zu schaffen, um standardisierte Qualitätsstandards für die rheumatoide Arthritis [14] und axiale Spondyloarthritis [15] über die Kerndokumentation zu erfassen. Dies würde das Qualitätsmanagement der teilnehmenden Zentren über die jährliche Einrichtungsauswertung weiter aufwerten.

Perspektivisch ist der Verzicht auf den Versand und die Erfassung von Fragebögen in Papierform wünschenswert. Für Einrichtungen, denen die Nutzung von Tablets nicht möglich ist, wird es jedoch noch die Möglichkeit der papierbasierten Erfassung geben. Durch die Umstellung auf ein einheitliches Dokumentationssystem reduziert sich der zeitliche Aufwand für die Fehlerprüfung und Datensatzerstellung im DRFZ erheblich, sodass angestrebt wird, die Einrichtungsauswertungen und die Standardpräsentation für das abgelaufene Jahr bereits im Folgejahr ausgeben zu können.

Auch die Register des DRFZ sollen perspektivisch mit RheMIT Daten erheben. Für alle Einrichtungen, die für die Register und die Kerndokumentation Daten erheben, könnte somit eine Doppelerfassung entfallen.

## Welchen Mehrwert hat die Teilnahme an der Kerndokumentation für eine Einrichtung?

Teilnehmende Einrichtungen erhalten jedes Jahr eine individuelle Einrichtungsauswertung, mittels derer sie ihre eigene Einrichtung mit anderen Einrichtungen der gleichen Versorgungsstufe (Universitätsklinikum, rheumatologische Klinik/Praxis) vergleichen können und damit zu ihrem internen Qualitätsmanagement beitragen können. Einrichtungen mit hohen Fallzahlen sind an Publikationen beteiligt, alle anderen werden in den Publikationen aufgeführt. Sie sind an einem zentralen Projekt der rheumatologischen Versorgungsforschung beteiligt. Die Kerndokumentation ist eine wichtige Informationsquelle für DGRh, BDRh und die Deutsche Rheuma-Liga, zudem beliefert sie die Gesundheitsberichterstattung des Bundes mit Daten. Die Daten der Kerndokumentation sind eine wichtige Grundlage für gesundheitspolitische Entscheidungen. In begrenztem Umfang stehen Mittel zur Vergütung der Dokumentation zur Verfügung. Entscheidend ist aber für alle teilnehmenden Einrichtungen die Idee, mit ihrer Datensammlung dieses für die deutsche Rheumatologie entscheidende Projekt mitzutragen.

## Ansprechpartner:innen

DRFZ-Ansprechpartnerinnen für eine Kontaktaufnahme sind Dr. Johanna Callhoff (Studienleitung), Dr. Katinka Albrecht (medizinische Leitung) und Katja Thiele (Projektkoordination). Bei Interesse an einer Teilnahme steht das Team Kerndokumentation im DRFZ (kerndokumentation@drfz.de) zur Verfügung. Das DRFZ schickt dann gern ausführliche Informationen zur Teilnahme oder vereinbart einen Termin, um Fragen zu besprechen.

## Fazit für die Praxis

Nachdem die anfänglichen Hürden in der Umstellung der Kerndokumentation auf RheMIT bewältigt sind, ist jede weitere Praxis oder Klinik, die an der Kerndokumentation teilnehmen möchte herzlich willkommen. Jede neue Einrichtung ermöglicht eine präzisere Abbildung der rheumatologischen Versorgung in Deutschland. Der Vergleich der Versorgungsstufen kann ausgebaut und für das Qualitätsmanagement der deutschen rheumatologischen Versorgung genutzt werden.
